# New Approach for the Determination of Radiological Parameters on Hardened Cement Pastes with Coal Fly Ash

**DOI:** 10.3390/ma14030475

**Published:** 2021-01-21

**Authors:** Ana María Moreno de los Reyes, José Antonio Suárez-Navarro, Maria del Mar Alonso, Catalina Gascó, Isabel Sobrados, Francisca Puertas

**Affiliations:** 1 Department of Materials, Eduardo Torroja Institute for Construction Sciences (IETcc-CSIC), 28033 Madrid, Spain; ana.moreno@ietcc.csic.es (A.M.M.d.l.R.); mmalonso@ietcc.csic.es (M.d.M.A.); 2 Department of Environment, Environmental Radioactivity and Radiological Surveillance (CIEMAT), Avenida Complutense 40, 28040 Madrid, Spain; ja.suarez@ciemat.es (J.A.S.-N.); catalina.gasco@ciemat.es (C.G.); 3 Department of Energy, Environment and Health, Institute of Material Sciences of Madrid (ICMM-CSIC), 28049 Madrid, Spain; isobrado@icmm.csic.es

**Keywords:** Portland cement, fly ash, SCMs, NORM wastes, characterization, gamma-ray spectrometry

## Abstract

Supplementary cementitious materials (SCMs) in industrial waste and by-products are routinely used to mitigate the adverse environmental effects of, and lower the energy consumption associated with, ordinary Portland cement (OPC) manufacture. Many such SCMs, such as type F coal fly ash (FA), are naturally occurring radioactive materials (NORMs). ^226^Ra, ^232^Th and ^40^K radionuclide activity concentration, information needed to determine what is known as the gamma-ray activity concentration index (ACI), is normally collected from ground cement samples. The present study aims to validate a new method for calculating the ACI from measurements made on unground 5 cm cubic specimens. Mechanical, mineralogical and radiological characterisation of 28-day OPC + FA pastes (bearing up to 30 wt % FA) were characterised to determine their mechanical, mineralogical and radiological properties. The activity concentrations found for ^226^Ra, ^212^Pb, ^232^Th and ^40^K in hardened, intact 5 cm cubic specimens were also statistically equal to the theoretically calculated values and to the same materials when ground to a powder. These findings consequently validated the new method. The possibility of determining the activity concentrations needed to establish the ACI for cement-based materials on unground samples introduces a new field of radiological research on actual cement, mortar and concrete materials.

## 1. Introduction

Concrete, whose main component or binder is Portland cement, is the most widely used construction material in the world. Such extensive use necessitates the manufacture and consumption of vast amounts of Portland cement. Worldwide output, which stood at 4200 million tons in 2019 [1], is expected to reach 6000 million tons by 2050 [2,3]. Portland cement manufacture is associated with a number of adverse effects, including high (essentially thermal and electric) power consumption, extraction and use of large amounts of natural resources such as limestone, clay, sand and fossil fuels, and the emission of enormous amounts of greenhouse gases, CO_2_ among them. Cement manufacture is estimated to account for around 8% of total anthropogenic CO_2_ released into the atmosphere [4,5,6,7,8].

Such adverse effects have driven the study and use of industrial waste and by-products from widely varying industries for cement and concrete manufacture. The aims of such practice include achieving the sustainability goals set out in the UN’s Agenda 2030 and implementing the guidelines for what is known in the EU as the circular economy [9,10], i.e., to favour more sustainable construction [4,8,9,11,12,13,14,15,16]. Some of the industrial waste and by-products apt for cement and concrete manufacture are members of the family of so-called NORMs (naturally occurring radioactive materials). Inasmuch as those materials are characterised by significant natural radionuclide content, the excess dose of gamma-ray activity released when they are used in civil and building construction must not be ignored [10]. In other words, the inclusion of NORMs in cement and concrete should not pose any added environmental or public health problems.

The external gamma radiation released by building materials in ways affecting the population at large must be assessed as laid down in European Directive 2013/59/EURATOM [17]. Article 75, item 2 of the directive defines a screening tool known as the activity concentration index (ACI) for gamma radiation emitted by building materials [4,18]. It also stipulates that to ensure their safe radiological use, these materials should not exceed an effective dose of 1 mSv a^−1^ [19] above the natural background, which is estimated at 50 nGy h^−1^ in Europe. The ACI is calculated from Equation (1):

(1) $$\mathrm{ACI}=\frac{C_{226_{\mathrm{Ra}}}}{300}+\frac{C_{232_{\mathrm{Th}}}}{200}+\frac{C_{40_{K}}}{3000}$$

where $C_{226_{\mathrm{Ra}}}$,${\;C}_{232_{\mathrm{Th}}}{\mathrm{and}\;C}_{40_{K}}$ are, respectively, ^226^Ra, ^232^Th and ^40^K activity concentration in the building material.

The ^226^Ra, ^232^Th and ^40^K activity concentrations in building materials, with or without industrial waste/by-products and whether anhydrous or hydrated, are normally determined with gamma-ray spectrometry on a powdery sample of the material to be analysed [4,20].

A new method developed by the authors of this paper determines the activity concentration of those radionuclides with great accuracy and precision and calculates the ACI on intact (=unground) samples of hardened Portland cement paste [21]. This new procedure can be described briefly as follows: 5 cm cubic (type I, i.e., without additions) Portland cement specimens were prepared and allowed to harden. The respective energy efficiency curve was calculated (in that study with LabSOCS modelling software) and validated on the intact 5 cm cubic specimens using different gamma-ray emitters ranging from 46.5 keV (^210^Pb) to 1460 keV (^40^K). The resulting gamma cocktails were then uniformly blended with water and cement. Gamma-ray spectrometry analyses were run on compact solid specimens 48 h and 64 days after the mixes are prepared. The specimens were measured at different heights with a (in the aforementioned study, LabSOCS-characterised) high purity germanium (HPGe) detector. According to the results: (i) the experimentally tested activities found for the six sides of the hardened cubic specimens (using LabSOCS-calculated efficiency) were statistically comparable; (ii) the variation in efficiency with quadratic specimen height satisfactorily corrected the gamma photon attenuation induced by the photoelectric and Compton effects in the energy range studied (46.54 keV to 1460 keV), as well as changes in the solid angle of the detector; (iii) as the activity found on the six sides of the intact specimens was statistically indistinguishable, and such activity could be determined by measuring just one side; (iv) the accuracy and precision observed for the geometry proposed met the standard acceptability criteria applied in environmental radioactivity laboratories; and (v) no significant differences were found between 48 h and 64 days paste activity in the intact specimens.

As this new method is intended for application to real samples (which bear no added gamma cocktails), it has to be validated by measuring the activity concentration of the radionuclides in the NORM waste. Unlike the cubic samples in the earlier study, radionuclides form part of the structure of such waste and therefore must be examined to see if their determination is affected by hydration reactions. The NORM waste chosen in this case was a type F fly ash (FA), a mineral addition or supplementary cementitious material (SCM) routinely used in cement and concrete manufacture [10].

Fly ash, an industrial by-product generated in coal-fired steam power plants used as a mineral addition, is characterised by the high activity concentration of its natural radionuclides [10]. The natural radioactivity present in FA depends on the geological origin of the coal used and the ash particle size [22,23,24]. US Geological Surveys show that coal containing phosphate minerals such as monazite or apatite has high radioactive ^232^Th concentrations, whereas coal containing both organic matter and mineral fractions has high ^238^U radioactive series contents [25]. When fly ash is mixed with Portland cement, the activity concentration index (ACI) in the resulting OPC + FA blends rises due to the increased presence of the gamma-ray emitting radionuclides used to calculate the index. The theoretical ACI values based on aluminium- or calcium-sulphate fly ash range from 1.2 to 1.3 [10,11,20,21,24,26,27], compared to pure Portland cement values of 0.2 to 0.3 [25,28,29,30,31].

As noted, in the previous study the exact concentrations of the gamma cocktail components used to prepare cement pastes were determined separately, not inside the actual matrix. This study aims to verify the technological validity of the new method using real building materials (OPC + FA) in which the radionuclides form part of the matrix, as they are present in the starting Portland cement and type F fly ash. The purpose is to validate the new method for measuring radionuclide activity concentration and determine the ACI on intact Portland cement pastes with a variable type F fly ash content. The procedure consisted in preparing 5 × 5 × 5 cm^3^ specimens of OPC pastes bearing FA at replacement ratios of 0 wt % to 30 wt % and subsequently determining their mineralogical composition and microstructure as well as the ^40^K, ^226^Ra and ^212^Pb (^232^Th) activity concentrations in the materials. All the analyses were run on 28-day specimens or ground samples, as the prior study showed that whilst the radionuclide concentration values recorded are unaffected by hydration and/or hardening time, readings should advisably be made after most of the chemical hydration reactions have run their course.

The partial objectives sought in the study include:

(1) Mechanical, mineralogical and structural characterisation of OPC + FA pastes
(2) Determination of the radionuclide activity concentrations and ACI in both the anhydrous components and intact 28-day pastes
(3) Validation of the method, comparing the radionuclide concentration and ACI values for the intact (unground) specimens and the ground samples of the same mixes.

## 2. Materials and Methods

The materials used and tests conducted in pursuit of the primary aim of the present study and in line with the methodology described in the Introduction are discussed below.

### 2.1. Materials

The chemical composition of CEM I 52.5 R (OPC) and type F (as defined in ASTM (American Society for Testing and Materials) standard C618-03) fly ash used in this study is given in Table 1. Chemical composition was determined on a Bruker S8 Tiger XRF spectrometer (Billerica, MA, USA), whilst loss on ignition (LoI) and insoluble residue (IR) were established as recommended in European standard EN 196-2:2014 [32].

**Figure 1:**
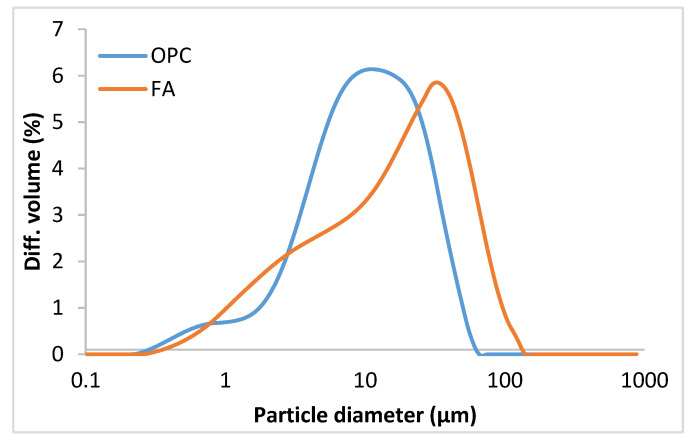
OPC and FA particle size distribution.

The particle size distribution of both materials, determined by laser diffraction on a Mastersizer analyser fitted with an He-Ne 632.8 nm laser, is listed in Table 2 and graphed in Figure 1**.** The Blaine specific surface of the two starting materials was found as recommended in standard EN 196-6:2010 [33].

Quantitative and qualitative mineralogical composition for OPC and FA were recorded with a Bruker AXL Advance D8 diffractometer (Billerica, MA, USA) fitted with an ultrafast Lynxeye X-ray detector and a 2.2 kW copper anode, configured for use without a monochromator. The mineralogical phases detected were quantified with Rietveld analysis using DIFFRAC-EVA.V4.2 software and the Crystallography Open Database (COD). The vitreous phase content in FA, 62.09 wt %, was determined with a selective 1% HF attack [34]. The quantitative XRD findings for the two materials are given in Table 3.

### 2.2. Preparation of Anhydrous OPC + FA Blends and Hydrated Pastes

The five OPC + FA prepared blends differed in the FA replacement ratio, which ranged from 0 wt % to 30 wt %. The raw materials were blended for 90 min in a mechanical blender-mixer. The proportion of FA in the OPC+FA blends and liquid/solid (l/s) ratios used to prepare the pastes are given in Table 4. The pastes were tested for plasticity and consistency as described in the European standard EN 196-3 [35], to ensure comparability.

**Figure 2:**
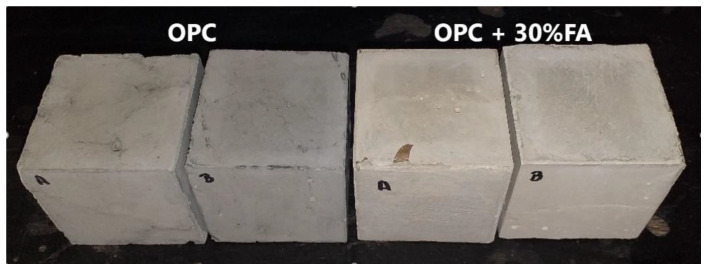
Five cm cubic specimens bearing 100%OPC and 70%OPC + 30%FA.

Five-centimetre cubic specimens were prepared from all the blends listed in Table 4 as specified in EN 197-1:2018 [36] and cured for 28 days in a moist cabinet at a relative humidity of 99% and a temperature of 21 ± 2 °C. A photograph of the fully cured OPC + 30%FA specimens is reproduced in Figure 2.

### 2.3. Tests Conducted

Prior to testing, hydration was detained in the 28-day intact specimens by soaking in isopropanol for 48 h. The cubes were subsequently dried for approximately 5 min under an infrared lamp and then stored in a vacuum desiccator for several days to eliminate moisture and any isopropanol residue. These samples were then tested as described below.

(a) Determination of compressive strength (three specimens per mix) on an Ibertest Autotest (Madrid, Spain) 15,000 kN test frame as recommended in European standard EN 196-2:2014 [32].
(b) Determination of specimen porosity and water sorptivity. Total porosity was determined on a Micromeritics Autopore IV 9500 analyser (Norcross, GA, USA) designed for pressures of up to 36,000 psi, equivalent to a pore size of 0.0067 µm. Water sorptivity and density were found as described in European standard EN 12390-7:2009 [37].
(c) Paste mineralogical and structural characterisation. Differential thermal and thermogravimetric analyses were conducted to determine paste mineralogy on a TA Instruments Q600 TGA-DSC-DTA. (New Castle, DE, USA) The test was run on platinum crucibles, ramping temperature at 10 °C/min from the ambient 25 ± 2 °C to 80 °C. After 1 h at the latter, temperature was raised at the same ramping rate to 900 °C. The entire test was conducted in a nitrogen atmosphere, flowing at 100 mL/min. ^29^Si MAS-NMR was recorded with a high-resolution solid-state Bruker Avance 400 spectrometer (Rheinstetten, Germany) equipped with a Fourier transform unit, at 79.49 MHz (9.4 T magnetic field) while spinning (10 kHz) the sample at a 54° 44” magic angle. A 6 µs pulse and 10 s recycle delay were used to maximise the intensity of the experimental signal. A total of 800 accumulations were acquired. Chemical shifts (δ, in ppm) were measured relative to tetra-methyl-silane (TMS, for ^29^Si). Spectra were fitted to Gaussian and Lorentzian peak shapes by applying non-linear least-squares iteration with Winfit software (Bruker). The ^29^Si MAS-NMR findings were used to find the degree of reaction (D.R.) in the Portland cement + FA blends with Equation (2) and the mean chain length (MCL) of the primary reaction product (C-S-H gel) from Equation (3) below:
  (2) $$D.R.=100-Q^{0}$$
  (3) $$\mathrm{MCL}=\left[Q^{1}+Q^{2}+\frac{3}{2}Q^{2}\left(1\mathrm{Al}\right)\right]/0.5\cdot Q^{1}$$
  where the Q^0^ units (−70 ppm to −74 ppm) refer to the monomeric signals (SiO_4_^4−^) generated by alite and belite, the anhydrous phases in the cement; and Q^1^ (−75 ppm to −81 ppm) to the dimeric units in C-S-H gel which, like the Q^2^ (1Al) units with a signal at −82 ppm, may indicate the presence of Al which replaces Si atoms in bridging sites. The interlayer uptake of alkaline or Ca^2+^ ions would balance the charges. The −82 ppm signal was disregarded in this study, given the overlap with the Q^1^ and Q^2^(0Al) (at around −85 ppm) signals, the latter denoting the presence of tetrahedral Si in bridging sites.
(d) Radiological characterisation of cement pastes as intact cubic specimens and solid ground samples. Natural radionuclide activity concentration in the pastes was measured with gamma-ray spectrometry using two high purity germanium detectors: one featuring Pb-shielding and coaxial extended range detection and the other Fe-shielding and a broad energy system. The specifications for each are listed in the Supplementary Information, Supplementary S1 (Table S1). The electronic chain associated with the detectors included a Canberra Industries amplifier and the same firm’s ethernet acquisition interface module (AIM) analogue to digital converter. The spectra were analysed using their Genie 2000 software. ^226^Ra, ^232^Th (^212^Pb), and ^40^K gamma-ray emissions were identified on the grounds of their respective characteristic photo peaks at 186 keV (^226^Ra), 238 keV (^212^Pb) and 1460 keV (^40^K). ^235^U interference in the ^226^Ra 186 keV photo peak and ^228^Ac interference in the 1460 keV photo peak were eliminated by applying the algorithm developed in [38]. The energy efficiency curves for the 76 mm ∅, tall plastic dishes and 30 mm high plastic Petri dishes containing the ground samples as well as for the intact 5 cm cubic specimens were calculated for each type of sample (anhydrous; intact, hydrated 5 cm cubic specimens prepared with the various Portland cement blends; and the respective ground samples) with Canberra Industries LabSOCS software applying a procedure described elsewhere by the authors [21]. Three types of samples were analysed:
  (i) Anhydrous OPC and FA powders with a maximum particle size of 63 µm
  (ii) Intact, 5 cm cubic OPC paste specimens blended with FA at the five replacement ratios listed in Table 4 (top and bottom measurements)
  (iii) Powder samples with a particle size of under 63 µm, obtained by grinding the OPC + FA cubic paste specimens described in (ii).

Powders (i) and (iii) were dried to a constant weight and packed into plastic Petri dishes. After hydration was detained as described above, the intact cubic specimens (ii) were weighed (P_0_) and measured (H_0_) and subsequently sealed with an epoxy resin to prevent ^222^Rn diffusion. All six sides of the specimens were coated with the resin, after mixing its two components (A and B), with chemical compositions C_30_H_43_O_7_Cl and C_17_H_30_ON_2_, at a ratio of 100/60 for 1 min. Two coats of resin were applied, drying the samples in an oven at 40 °C for 2 h after each application. The weight (P_1_) and height (H_1_) of the specimens measured after applying the second coat were the values subsequently used in LabSOCS simulations (Version 4.4.1). The levels of natural and artificial radionuclides in the resin used were found to be lower than the radioactive background.

**Figure 3:**
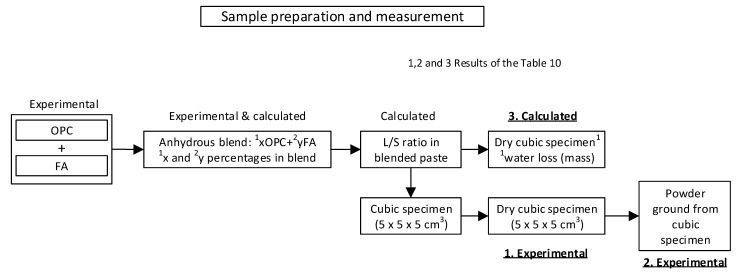
Sample preparation and measurement sequence for gamma-ray spectrometric analysis.

Specimen and ground sample preparation and gamma-ray spectrometric analysis were conducted as sequenced in Figure 3. The experimental gamma-ray spectrometric readings taken on (i) intact dried specimens and (ii) the respective ground samples were compared to the values calculated with the model for each blend based on the mass loss listed in Table 6 (primarily attributable to water evaporation between 25 °C and 81.5 °C). The theoretical ACI was calculated for the fresh pastes as well as for the pastes assuming the mass loss given in Table 6.

The activity concentrations obtained for the type (i) samples were used to calculate the theoretical activity concentrations for both the intact cubic (type (ii)) specimens and the respective ground (type (iii)) samples with Equation (4):

(4) $$A_{T}=\frac{A_{\mathrm{AP}}\cdot \left(\frac{1}{1+l/s}\right)}{\left(1-w_{L}\right)}$$

where A_AP_ is the activity concentration for ^226^Ra, ^212^Pb or ^40^K (in Bq·kg^−1^) obtained from the solid ground (type (i)) samples, l/s is the water-cement ratio used to prepare the hardened OPC + FA blended cements and w_L_ is the water loss expressed as a decimal. The uncertainty associated with the theoretical activity found (u(A_T_), Bq·kg^−1^) was calculated as per Equation (5):

(5) $$u\left(A_{T}\right)=\left(\frac{1}{1-w_{L}}\right)\cdot \left(\frac{1}{1+l/s}\right)\cdot u\left(A_{\mathrm{AP}}\right)$$

The uncertainty associated with the ACI calculated using Equation (1) was estimated as

(6) u(ACI)=(1300)2·u2(AR 226a)+(1200)2·u2(AP 212b)+(13000)2·u2(AK 40)

where u2(AY X) is the activity concentration uncertainty for ^226^Ra, ^212^Pb or ^40^K (in Bq·kg^−1^).

The uncertainty of the weighted average, in turn, was found further to the Bambynek criterion [39] as the higher of the external ($u{\left(\overset{¯}{A}\right)}_{\mathrm{ext}}$) or internal ($u{\left(\overset{¯}{A}\right)}_{\mathrm{int}}$) uncertainty (Equations (7) and (8) respectively), calculated for a coverage factor k = 1.

(7) $$u{\left(\overset{¯}{A}\right)}_{\mathrm{ext}}=\sqrt{\frac{\sum_{i=1}^{N}u{\left(A\right)}_{i}\cdot {\left(A_{i}-\overset{¯}{A}\right)}^{2}}{\frac{N}{N-1}\cdot \sum_{i=1}^{N}u{\left(A\right)}_{i}}}$$

(8) $$u{\left(\overset{¯}{X}\right)}_{\mathrm{int}}=\sqrt{{\left(\overset{N}{\underset{i=1}{\sum }}u{\left(A\right)}_{i}^{-2}\right)}^{-1}}$$

where $A_{i}$ is the i-th activity concentration, $u{\left(A\right)}_{i}$ is the uncertainty of the i-th activity concentration and N is the number of values.

The theoretical activity calculations for ^40^K are given by way of example in the Supplementary Information, Supplementary S3 [40]. More information about all the sample calculations is presented in Supplementary S4.

## 3. Results and Discussion

The most prominent findings and their interpretation in connection with the partial objectives defined in the introduction are described in this section, which summarises paste mechanical, mineralogical and structural characterisation as well as the results of the gamma-ray radiological exploration. All the analyses were conducted on both anhydrous OPC and FA samples as well as on the hydrated and hardened 28-day humidity chamber-cured specimens. 

### 3.1. Paste Mineralogical and Structural Characterisation. Mechanical Performance.

Further to the data listed in Table 5, only the 28-day pastes with FA contents of over 20% exhibited slightly lower compressive strength than the reference OPC. Such declines in mechanical strength in fairly early age (28 days) pastes with high FA contents have been reported by other authors [41,42].

A correlation was observed between specimen strength and both total porosity and water sorptivity (see Table 5). The scant differences between pastes in the former confirmed the similarity in all the materials of the water sorptivity values (which afford an indication of capillary porosity) and Hg porosimetry-determined total porosity.

The TG mass loss findings for the 28-day OPC + FA pastes are given in Table 6, whilst the DTA/TG readings for all the OPC + FA pastes studied are shown in the Supplementary Information, Supplementary S2 (see Figure S1).

The temperature ranges shown in Table 6 were associated with the heat-induced physical-chemical changes observed in the pastes. The first range (25 °C to 81.5 °C) was indicative of physiosorbed (=unbound) water [43]; the second (81.5 °C to 350 °C) of partial or total C-S-H gel and ettringite dehydration [44]; the third (350 °C to 550 °C) essentially of portlandite de-hydroxylation [45]; and the fourth (550 °C to 900 °C) primarily of decarbonation of the limestone present in the cement (see Table 3) and the calcium carbonates generated as a result of partial paste weathering [46].

An analysis of the data in Table 6 revealed that in the temperature range studied (25 °C to 900 °C), rising FA content was associated with a slight decline in the percentage of mass loss relative to the OPC reference. That finding could be explained by the lower OPC content in the blends and lower 28-day FA reactivity. Nonetheless, the practically constant loss in the 350 °C to 550 °C range would denote the lack of any substantial change in Ca(OH)_2_ content in the cements, an indication of a certain hastening of OPC hydration in the presence of FA [47,48,49]. The findings delivered by this technique attested overall to the scant mineralogical alterations taking place in the pastes with FA contents of up to 30% (by cement weight).

^29^Si MAS-NMR was conducted to characterise paste structure. The deconvoluted data for the ^29^Si MAS-NMR spectra are given in Table 7 and the deconvoluted spectra are reproduced in the Supplementary Information (Supplementary S2, Figure S2). The parameters calculated from the deconvoluted data are listed in Table 8.

The ^29^Si MAS-NMR spectrum for the anhydrous OPC (Supplementary Information, Supplementary S2, Figure S2A) could be deconvoluted into four signals indicative of tetrahedrally coordinated Si (SiO_4_^4−^, Q^0^ monomeric units) [50,51,52] attributable to the anhydrous phases alite and belite present in OPC. The signal at around −71 ppm and a low intensity signal at −74 ppm were generated by belite (which can be likened to β-C_2_S) and two wider signals at approximately −70 ppm and −73 ppm by alite (C_3_S). The six signals identified on the deconvoluted spectrum for the FA used in this study, (reproduced in Supplementary Information, Supplementary S2, Figure S2B), were assigned as follows: at −89 ppm, to the Q^3^ units in mullite; at −97 ppm, to Q^3^(Al) units [53]; at −104 ppm, to Q^3^(4Al) units; and at −110 ppm and −118 ppm, to Q^4^(0Al) units assigned to the quartz present in the fly ash [54]. The sixth, a wide signal, was attributed to a vitreous phase mineral.

Supplementary Information, Supplementary S2, (Figure S2C–G) reproduce the deconvoluted ^29^Si MAS-NMR spectra for the 28-day OPC + FA pastes. Two peaks were observed, one centred at a chemical shift of around −79 ppm, associated with Q^1^ units, and the other at −84 ppm, with Q^2^(0Al) units. Q^2^(1Al) units, which would be located at a shift of around −82 ppm, were disregarded in the fitting because they overlapped with Q^1^ and Q^2^(0Al) units. Simulation of the experimental spectra based on the peak areas for each signal and the total spectrum area yielded the percentage of each Q^n^ unit present in the C-S-H gel. Those percentages were then used to calculate the total degree of paste hydration or total chain length further to Equations (2) and (3), listed in Table 8. According to the data in that table, the amount of hydration product in the pastes did not vary substantially with increasing FA content. The technique revealed the presence of pozzolanically unreacted FA in the 28-day samples bearing >20%FA. The degrees of reaction delivered by the technique (Table 8) exhibited no significant variations except in the sample with 30%FA, the explanation for which would require further study. According to the ^29^Si MAS NMR findings, the presence of up to 30%FA in the cement did not significantly alter the composition of the primary reaction products. Those findings were consistent with the physical and mechanical data given in Table 5 (in particular, density, sorptivity and total porosity).

### 3.2. Radiological Characterisation of the Hardened Intact Specimens and Solid Powder Samples

**Figure 4:**
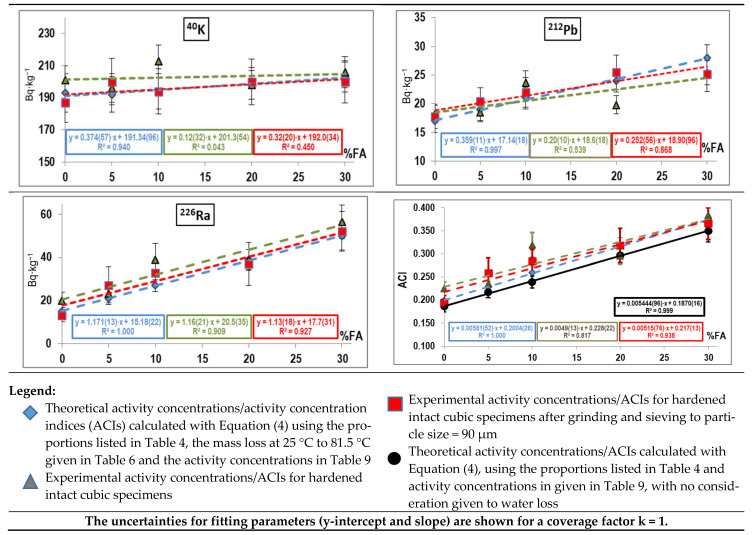
^226^Ra, ^232^Th (^212^Pb) and ^40^K activity concentrations and theoretical ACIs for: intact cubic specimens considering and disregarding water loss; experimental ACIs for intact cubic cement paste specimens; and experimental ACIs for powders obtained by grinding intact specimens to a particle size of 90 µm.

The ^226^Ra, ^212^Pb and ^40^K activity concentrations for the OPC and FA powders, measured separately, as well as for the anhydrous OPC + FA blends used to prepare the hardened cement pastes, are listed in Table 9. The counting geometry validation findings, in turn, are graphed in Figure 4 and the respective values given in Table 10. The theoretical activity concentrations for the intact cubic specimens were calculated by entering the data in Table 9, the proportions in Table 4 and the water loss values at 25 °C to 81.5 °C listed in Table 6 into Equation (4). Those theoretical values were compared to the concentrations observed experimentally in both the intact cubic specimens and the respective ground samples. The ACIs, in turn, were compared to a second theoretical value, calculated under the assumption of no water loss (Equation (4) with w_L_ = 0). The uncertainty associated with the slope and y-intercept were determined with the linear regression procedure described in [55]. The calculations are described in Supplementary Information, Supplementary S4 (Table S2).

As the data in Table 10 show, relative uncertainties of <10% were obtained for radionuclide activity concentrations in the intact specimens (counting geometry), the respective ground samples and the aforementioned theoretical calculations. In other words, the activity concentrations found for the intact cubic specimens were statistically equal to the values observed for the respective ground samples. The FA content in the pastes appeared to induce no significant effects, although a linear relationship was observed between activity concentration and the FA replacement ratio (Figure 4).

The results set out above may be interpreted as follows. The deduction drawn from an analysis of the DTA/TG findings in Table 6 and the ^29^Si MAS-NMR results in Table 7 and Table 8 is that 28-day hydration was similar in all the OPC + FA pastes from the standpoint of reaction product identity and proportions. This translated into similar strength, density and porosity values, as per the data in Table 5. According to the ^29^Si MAS NMR results (Table 8), at FA contents of over 20% part of the FA in the 28-day blends had not yet reacted, i.e., the pozzolanic reactions had not run their full course. Nonetheless, the presence of the FA appeared to expedite OPC hydration, as the Ca(OH)_2_ content in the pastes did not decline despite the lower OPC content in the initial blend. Further support for that premise was provided by the TG findings, which showed no decline in the water bound to either the C-S-H gel or ettringite (Table 6, 81.5 °C to 350 °C and 350 °C to 550 °C). In other words, the hydrated phases did not differ substantially in the 28-day OPC + FA pastes bearing up to 30% FA (by cement weight), subsequently analysed with gamma-ray spectrometry.

The activity concentrations observed for the reference OPC were equivalent to the values reported in the literature [56]. Although the ^226^Ra concentrations in the FA were higher than the worldwide and European mean values for building materials [57,58], they were consistent with other earlier findings [13,26]. Those values infer that the FA used was sourced from lignite, a type of coal characterised by a high percentage of organic matter [22,59].

The regression lines for the ^226^Ra, ^212^Pb and ^40^K activity concentration %FA content curves for both the intact cubic specimens and respective ground samples (Figure 4 and Table 10) were statistically equal, for both the slopes and the y-intercepts overlapped for coverage factor k = 2. The slope for ^226^Ra was steeper than for ^212^Pb and ^40^K because its activity concentrations in FA and OPC differed widely. The flatter slopes for ^212^Pb and ^40^K would explain the low correlation coefficients observed. The findings revealed agreement between the experimental values (intact specimens and respective ground samples) and the theoretical concentrations determined from the anhydrous powders (Table 9). The experimental and theoretical ACI values were also observed to agree when water loss data were taken into consideration. The regression line relating theoretical ACI to FA content when water loss was disregarded was not statistically comparable to the other three graphs, however. Whilst the four ACI curves had statistically equal slopes, the y-intercept differed on the graph in which physiosorbed water (25 °C to 81.5 °C) was not taken into consideration. The inference is that water loss at 25 °C to 81.5 °C (Table 6) in the 28-day specimens must be taken into consideration when calculating ACI under the experimental conditions prevailing in this study. The findings showed that Portland cement pastes blended with less than 30% FA are radiologically safe.

They also attest to the technological validity of the new method for determining ^226^Ra, ^212^Pb (^232^Th) and ^40^K activity concentrations in intact 5 cm cubic cement specimens (bearing up to 30%FA in the binder). 

Characterisation of the 28-day hydrated pastes revealed no significant differences in total porosity, sorptivity or density (Table 5). The similarity in density between the OPC pastes and the materials bearing up to 20% to 30% fly ash might explain the radiological findings. Indeed, further to those results variations in cement fly ash content affected the values measured with one or the other type of samples more than differences in paste microstructure or composition.

The validity of the new methodology, with which the ACI of cement-based materials can be determined with no need for grinding, will be further assessed in future research. More specifically, studies will focus on its applicability to cement pastes with a higher fly ash content, bearing other SCMs or found in more complex systems such as cement mortar or concrete.

## 4. Conclusions

The most prominent conclusions that may be drawn from the present study include the following.

(1) This study validated a new method for determining ^226^Ra, ^212^Pb and ^40^K activity concentrations in intact 5 cm cubic specimens directly, i.e., with no need to grind the material as in present practice. The method was validated for cement pastes with variable (up to 30 wt %) amounts of a NORM waste, specifically type F coal fly ash, at 28 days of hydration.
(2) No physical, mechanical, mineralogical or structural differences were observed between pastes bearing unblended OPC and those made from blends with up to 20%FA. In other words, the hydrated phases did not differ substantially in composition or content in the 28-day OPC + FA pastes bearing up to 30%FA (by cement weight), subsequently analysed with gamma-ray spectrometry.
(3) The activity concentrations found for ^226^Ra, ^212^Pb and ^40^K in the hardened intact 5 cm cubic specimens were statistically equal to the values for the respective ground samples and to the theoretical concentrations calculated separately for each of the two components from the values observed for the respective anhydrous powders. Under the experimental conditions applied, 28-day unbound water loss had to be taken into consideration for the theoretical concentration calculations to yield accurate results. The findings showed Portland cement pastes blended with less than 30%FA to be radiologically safe.
(4) They also attested to the technological validity of applying the new method for determining ^226^Ra, ^232^Th (^212^Pb) and ^40^K activity concentrations in intact 5 cm cubic specimens made with cement paste bearing up to 30%FA. The possibility of determining the activity concentrations needed to establish the ACI for cement-based materials on solid, unground samples introduces a new field of radiological research on cements, mortars and concretes actually used in construction.

## Figures and Tables

**Table 1:** Ordinary Portland cement (OPC) and Fly Ash (FA) chemical composition (wt %).

Material	CaO	SiO_2_	Al_2_O_3_	Fe_2_O_3_	K_2_O	MgO	Na_2_O	P_2_O_5_	SO_3_	TiO_2_	Other	LoI ^1^	I.R. ^2^
**OPC**	64.47	20.29	5.67	2.35	0.97	0.84	0.11	0.14	2.91	0.24	0.17	2.97	1.07
**FA**	4.78	42.44	26.95	18.40	1.53	0.80	0.50	0.20	1.44	1.07	0.03	1.63	7.78

^1^ LoI: loss on ignition ^2^ I.R.: Insoluble residue.

**Table 2:** OPC and FA particle size distribution and Blaine specific surface.

Material	Dv_10_ (µm)	Dv_50_ (µm)	Dv_90_ (µm)	Blaine (m^2^/kg)
**OPC**	2.34	9.31	27.01	404.68
**FA**	1.93	16.13	51.54	451.87

**Table 3:** OPC and FA quantitative mineralogical composition (wt %).

**Phases**	**Alite**	**Belite**	**Tricalcium Aluminate**	**Ferrite Phase**	**Gypsum**	**Bassanite**	**Calcite**	
**OPC**	64.21	13.16	8.98	5.82	1.77	1.64	4.42	
**Phases**	**Amorphous Phase**	**Quartz**	**Mullite**	**Hematite**	**Magnesium Ferrite**	**Magnetite**	**Maghemite**	**Calcite**
**FA**	62.09	7.97	20.43	2.41	3.99	1.65	0.82	0.64

**Table 4:** FA content and liquid/solid ratios in the blended pastes.

Cement	%OPC	%FA	l/s
OPC	100	-	0.31
OPC + 5%FA	95	5	0.32
OPC + 10%FA	90	10	0.32
OPC + 20%FA	80	20	0.32
OPC + 30%FA	70	30	0.32

**Table 5:** Mechanical strength, water sorptivity, density and total porosity in the specimens studied.

Material	Compressive Strength (MPa)	Sorptivity (%)	Density (g/mL)	Total Porosity (%)
OPC	69.4 ± 0.6	28.89 ± 0.2	1.84	17.88
OPC + 5%FA	72.1 ± 2.1	30.48 ± 0.2	1.79	18.94
OPC + 10%FA	71.8 ± 2.6	30.08 ± 0.2	1.80	17.16
OPC + 20%FA	69.3 ± 1.5	31.18 ± 0.2	1.78	18.44
OPC + 30%FA	61.2 ± 0.7	31.17 ± 0.2	1.77	22.52

**Table 6:** TG-determined 28 days mass loss (wt %) in the pastes studied.

Material	25–81.5 °C	Total Loss 81.5–900 °C	81.5–350 °C	350–550 °C	550–900 °C
OPC	6.30	15.94	5.39	5.24	5.31
OPC + 5%FA	6.25	15.73	5.75	5.58	4.40
OPC + 10%FA	6.07	15.31	5.66	5.02	4.63
OPC + 20%FA	6.06	15.10	6.07	5.12	4.34
OPC + 30%FA	5.86	14.10	5.64	4.56	3.90

**Table 7:** Signal positions and intensities on deconvoluted ^29^Si MAS-NMR spectra for 28-day OPC + FA pastes.

Material	Q^0^				Q^1^	Q^2^(0Al)	Q^3^(3Al)	Q^4^(0Al)	
OPC	−70.66 ppm I = 26.24%	−71.76 ppm I = 9.39%	−73.49 ppm I = 29.93%	−74.30 ppm I = 1.20%	−79.69 ppm I = 20.05%	−84.81 ppm I = 13.19%	-	-	-
OPC + 5%FA	−70.66 ppm I = 29.69%	−71.60 ppm I = 10.29%	−73.49 ppm I = 24.78%	−74.12 ppm I = 0.78%	−79.35 ppm I = 22.49%	−84.34 ppm I = 11.96%	-	-	-
OPC + 10%FA	−70.66 ppm I = 28.73%	−71.65 ppm I = 9.79%	−73.49 ppm I = 27.22%	−73.86 ppm I = 1.03%	−79.88 ppm I = 19.97%	−84.69 ppm I = 13.27%	-	-	-
OPC + 20%FA	−70.66 ppm I = 28.89%	−71.65 ppm I = 9.81%	−73.49 ppm I = 23.13%	−74.02 ppm I = 1.15%	−79.56 ppm I = 18.06%	−84.33 ppm I = 11.74%	-	−105.48 ppm I = 7.23%	-
OPC + 30%FA	−70.66 ppm I = 14.38%	−71.79 ppm I = 7.52%	−73.80 ppm I = 22.67%	−73.49 ppm I = 0.94%	−81.56 ppm I = 20.64%	−87.66 ppm I = 12.11%	−94.80 ppm I = 6.46%	−101.0 ppm I = 3.57%	−108.5 ppm I = 11.73%

**Table 8:** Parameters calculated from deconvoluted ^29^Si MAS-NMR spectra for 28-day OPC + FA pastes.

Material	Q^0^	Q^1^ + Q^2^	Q^2^/Q^1^	FA (n.r.) ^(a)^	D.R. ^(b)^	**MCL ^(c)^**
OPC	66.76	33.24	0.66	-	33.24	3.32
OPC + 5%FA	66.54	34.45	0.53	−0	34.46	3.06
OPC + 10%FA	68.29	31.71	0.66	−0	33.23	3.33
OPC + 20%FA	62.98	29.80	0.65	7.23	37.02	3.30
OPC + 30%FA	45.51	32.75	0.59	21.71	54.50	3.17

(Q^0^): % anhydrous material; (Q^1^ + Q^2^): % hydration product. ^(a)^ FA (n.r.): unreacted FA; ^(^^b)^ D.R.: degree of reaction; ^(c)^ MCL: Mean Chain Length of C-S-H gel.

**Table 9:** ^226^Ra, ^212^Pb and ^40^K activity concentrations in the anhydrous powders studied.

Material	^226^Ra (Bq·kg^−1^)	^212^Pb (Bq·kg^−1^)	^40^K (Bq·kg^−1^)
OPC	19.0 ± 3.9	21.3 ± 3.2	235 ± 15
FA	164 ± 27	66.8 ± 7.6	292 ± 18
OPC + 5%FA	22.6 ± 4.7	23.3 ± 4.7	237 ± 15
OPC + 10%FA	30.7 ± 6.3	25.5 ± 3.8	241 ± 15
OPC + 20%FA	48.2 ± 8.2	29.9 ± 4.4	247 ± 15
OPC + 30%FA	64 ± 11	35.4 ± 4.3	251 ± 16

Uncertainties given for a coverage factor k = 2.

**Table 10:** ^226^Ra, ^232^Th (^212^Pb) and ^40^K activity concentrations (in Bq·kg^−1^) for the OPC + FA blends studied: comparison of findings for intact cubic specimens, the respective ground samples and the theoretically calculated values (taking chemically unbound water loss into consideration).

1. 5 cm Cubic (Intact) Specimens					2. Powder Sample from Intact Specimens				3. Theoretical Values			
Material	^40^K	^238^U Series	^232^Th Series	ACI	^40^K	^238^U Series	^232^Th Series	ACI	^40^K	^238^U Series	^232^Th Series	**ACI**
		^226^Ra	^212^Pb			^226^Ra	^212^Pb			^226^Ra	^212^Pb	
OPC	201 ± 9	20.0 ± 3.9	18.2 ± 1.5	0.225 ± 0.015	187 ± 12	13.2 ± 2.9	17.8 ± 2.1	0.195 ± 0.015	193 ± 12	15.4 ± 1.8	17.3 ± 2.0	0.199 ± 0.012
OPC + 5%FA	196 ± 9	23.3 ± 4.1	18.5 ± 1.5	0.236 ± 0.016	200 ± 14	27.0 ± 8.9	20.4 ± 2.4	0.259 ± 0.032	192 ± 11	21.0 ± 2.0	18.9 ± 1.9	0.229 ± 0.012
OPC + 10%FA	212 ± 10	38.7 ± 6.9	23.6 ± 2.0	0.320 ± 0.025	194 ± 14	32.8 ± 6.1	22.1 ± 2.6	0.284 ± 0.025	194 ± 11	26.8 ± 2.7	20.7 ± 1.9	0.260 ± 0.014
OPC + 20%FA	198 ± 9	38.9 ± 4.8	19.8 ± 1.6	0.295 ± 0.018	200 ± 14	370 ± 9.9	25 ± 3	0.318 ± 0.037	199 ± 10	38.5 ± 4.6	24.4 ± 2.0	0.316 ± 0.019
OPC + 30%FA	206 ± 11	56.8 ± 7.9	25.4 ± 2.2	0.384 ± 0.029	200 ± 13	52.0 ± 9.2	25 ± 3	0.366 ± 0.034	202.9±9.4	50.2 ± 6.6	28.1 ± 2.3	0.374 ± 0.025

Uncertainties given for a coverage factor of k = 2.

## Data Availability

The data presented in this study and supplementary information are available on request from the corresponding author.

## Supplementary Materials

The following are available online at https://www.mdpi.com/1996-1944/14/3/475/s1, Figure S1: TG (green) and DTA (blue) 28 days thermograms for pastes prepared with: (A) OPC, (B) OPC + 5%FA, (C) OPC + 10%FA, (D) OPC + 20%FA and (E) OPC + 30%FA, Figure S2: 28 days ^29^Si MAS NMR spectra for (A) anhydrous OPC; (B) anhydrous FA; and pastes prepared with (C) OPC; (D) OPC + 5%FA; (E) OPC + 10%FA; (F) OPC + 20%FA; and (G) OPC + 30%FA, Table S1: Specifications for the high purity germanium detectors used in this study, Table S2: Calculations.
