# Supplementary material for: New Approach for the Determination of Radiological Parameters on Hardened Cement Pastes with Coal Fly Ash
Source: Materials (Basel). 2021 Jan 21;14(3):475. doi: 10.3390/ma14030475 (PMC7864329; doi:10.3390/ma14030475)
Supplement: Supplementary file 1 [file materials-14-00475-s001.pdf]

materials

Article

Supplementary Information

New Approach for the Determination of Radiological Parameters on Hardened Cement Pastes with Coal Fly Ash

Ana María Moreno de los Reyes [1], José Antonio Suárez-Navarro [2], Maria del Mar Alonso [1], Catalina Gascó [2], Isabel Sobrados [3] and Francisca Puertas [1,*]

[1] Department of Materials, Eduardo Torroja Institute for Construction Sciences (IETcc-CSIC), 28033 Madrid, Spain; puertasf@ietcc.csic.es (F.P.); ana.moreno@ietcc.csic.es (A.M.M.d.l.R.); mmalonso@ietcc.csic.es (M.d.M.A.)
[2] Department of Environment, Environmental Radioactivity and Radiological Surveillance (CIEMAT), 28040 Madrid, Spain; ja.suarez@ciemat.es (J.A.S.-N.); catalina.gasco@ciemat.es (C.G.)
[3] Department of Energy, Environment and Health. Institute of Material Sciences of Madrid (ICMM-CSIC). 28049 Madrid, Spain; isbrado@icmm.csic.es
* Correspondence: puertasf@ietcc.csic.es

Supplementary S1. Specifications for the HPGe Detectors Used in This Study

Table S1. Specifications for the high purity germanium detectors used in this study.

Parameter	Detector 07	Detector 90
Model	GXI0022	BE50360
Type	Extended Range Coaxial	Broad Energy
Resolution at 1.33 MeV (keV)	2.04	1.84
Relative efficiency at 1.33 MeV (%)	115.7	48.0
Crystal diameter (mm)	84	80
Crystal length (mm)	72	30
Outer shielding (cm)1	15	15
Inner shielding-1st layer (Cu) (mm)	3.0	2.5
Inner shielding-2nd layer (Zn) (mm)	-	1.5
Inner space shielding (dm^3)	25	25
Shielding composition	Fe	Pb

Supplementary S2. Mineralogical and Microstructural Characterisation of Ordinary Portland Cement (OPC) + Fly Ash (FA) Pastes. DTA/TG Thermograms and ^{29}Si MAS NMR Spectra

- Thermograms

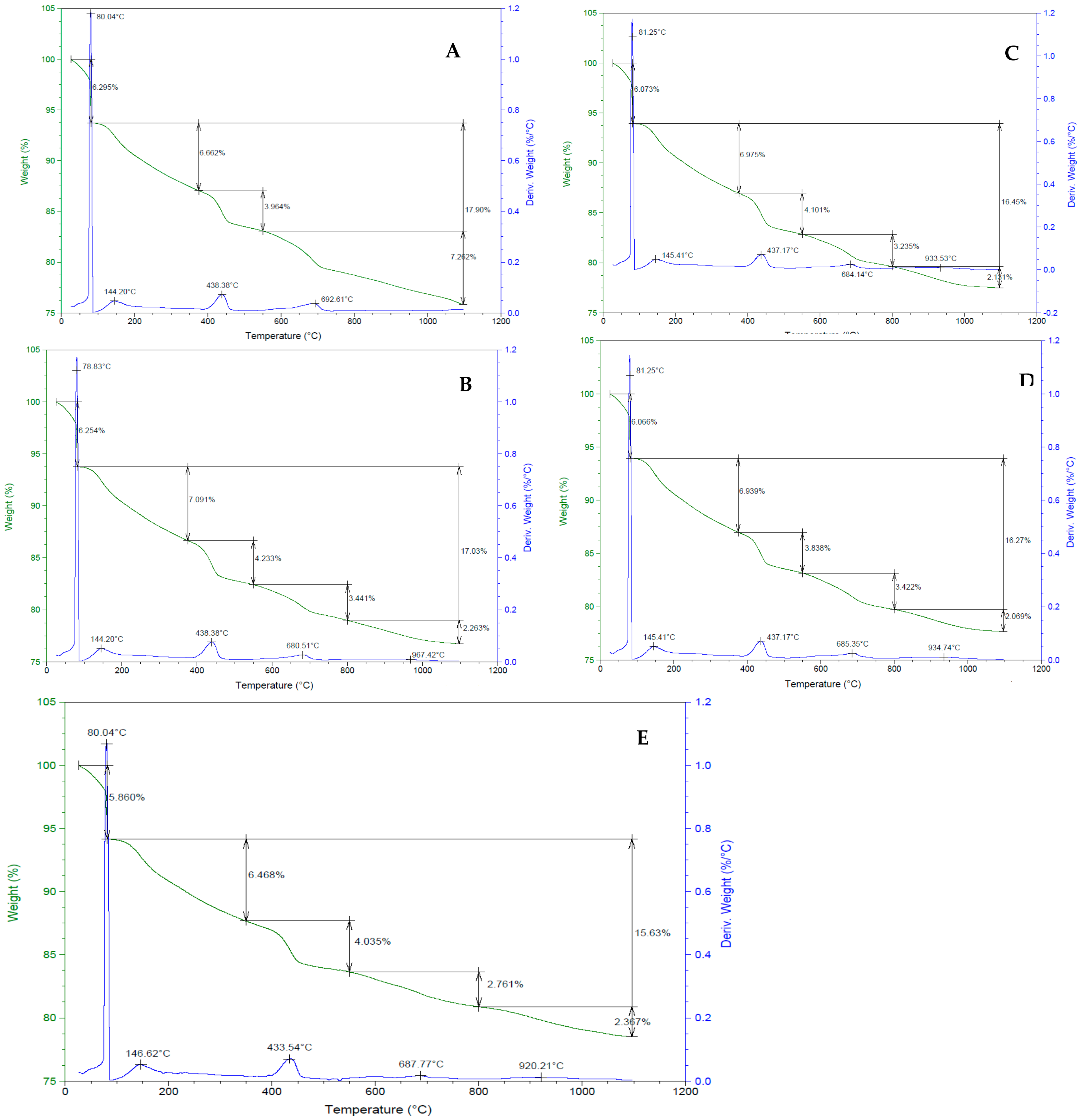

Figure S1. TG (green) and DTA (blue) 28 days thermograms for pastes prepared with: A) OPC, B) OPC + 5%FA, C) OPC + 10%FA, D) OPC + 20%FA and E) OPC + 30%FA.

- ^{29}Si MAS NMR spectra

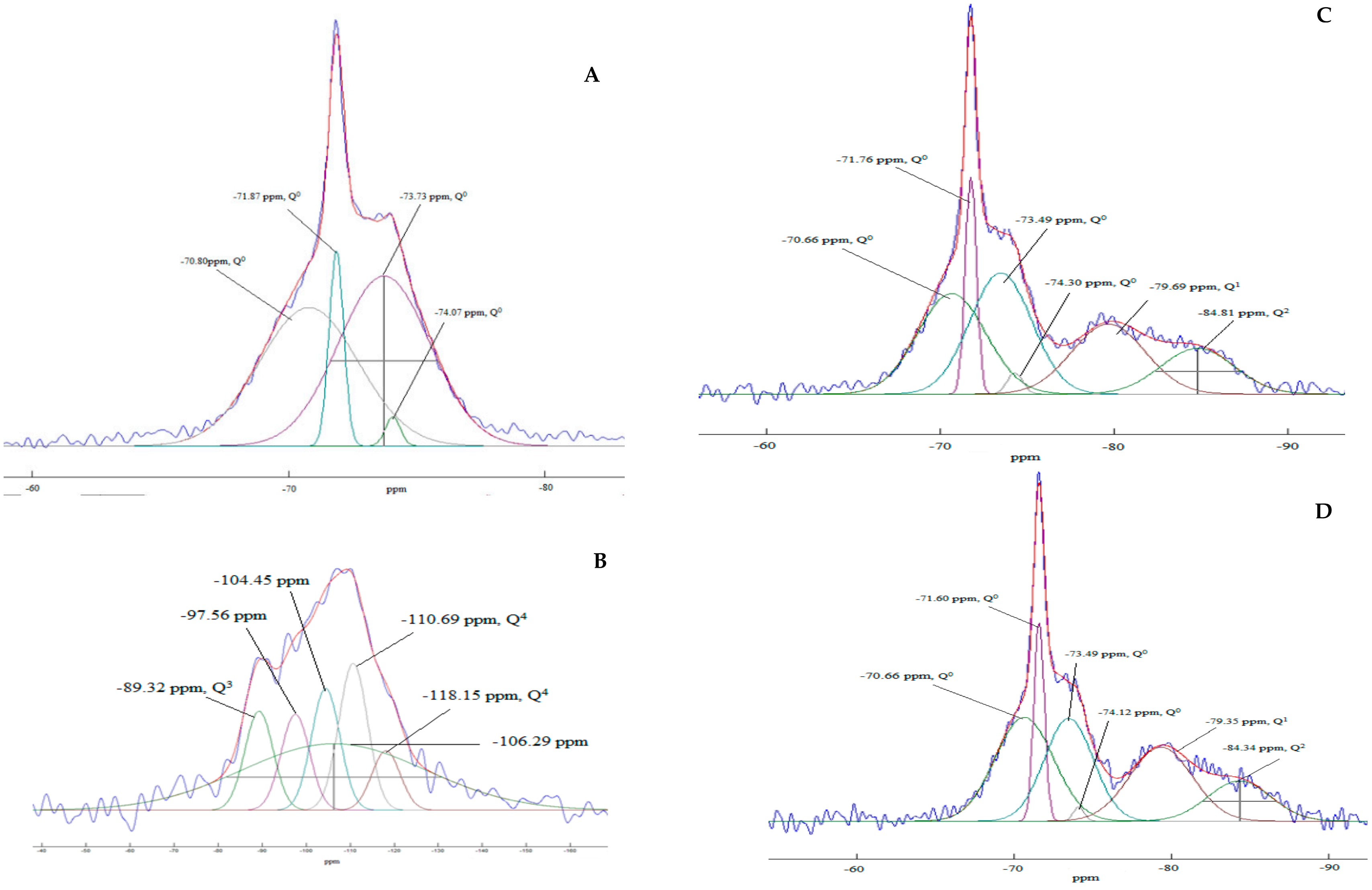

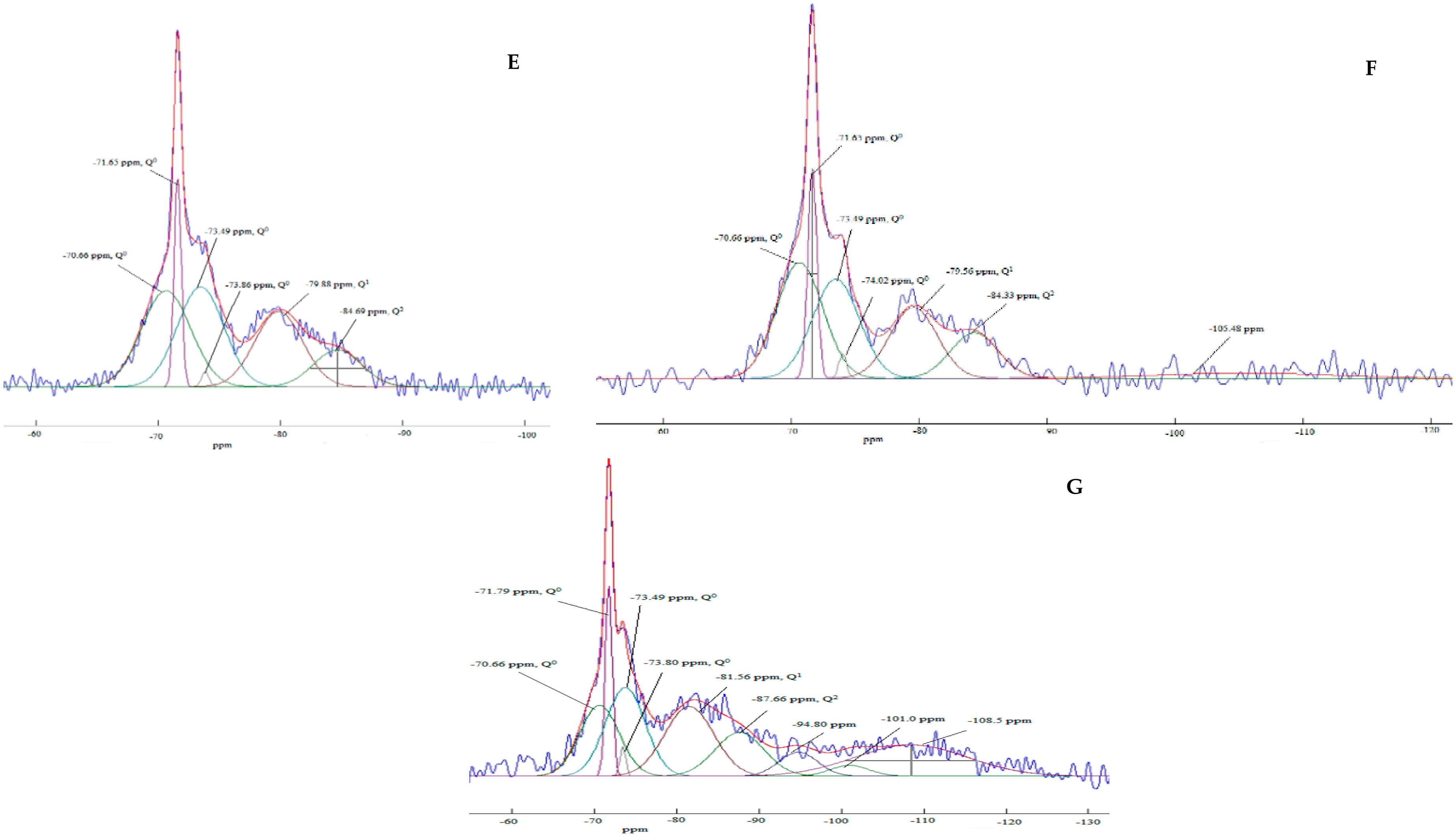

Figure S2. 28 days ^{29}Si MAS NMR spectra for A) anhydrous OPC; B) anhydrous FA; and pastes prepared with C) OPC; D) OPC + 5%FA; E) OPC + 10%FA; F) OPC + 20%FA; and G) OPC + 30%FA.

Supplementary S3. Comparison of Experimental and Theoretical Values

Gamma spectrometry measurements were taken on specimens before and after they were ground. The findings were compared to the theoretical values calculated for the blended paste before and after drying, assuming in the latter case the mass loss (at 25 °C to 81.5 °C), primarily attributable to water, as given in Table 6.

Supplementary S4. Theoretical Calculation of Radionuclide Activity Concentration in Anhydrous Starting Materials and Fresh and Dried Pastes and Associated Uncertainties

Theoretical calculation of activity concentration values (in Bq·kg^{-1}) and associated uncertainties (k = 2; 95 %) based on experimental measurements of natural radionuclides in unblended OPC and FA; and theoretical calculations for: anhydrous blends (used as example in Table S4. 1) blended pastes and blended pastes after loss of unbound water.

Table S2. Calculations.

Material	^{40}K	^{238}U series	^{232}Th series	ACI
		^{226}Ra	^{212}Pb	
FA[2]	292 ± 18	164 ± 27	66.8 ± 7.6	----
OPC[2]	233 ± 15	19.0 ± 4.7	21.3 ± 3.2	----
[1]OPC + 5%FA(blend)	236 ± 15*	26 ± 2.5	23.4 ± 2.4	0.283 ± 0.015
[1]OPC + 5%FA(paste)	180 ± 11	19.7 ± 1.9	17.7 ± 1.8	0.217 ± 0.012
[1]OPC + 5%FA(dry paste)	192 ± 11	21.0 ± 2.0	18.9 ± 1.9	0.229 ± 0.012

[1] Calculated values

* Differences between these values and those shown in the paper are due to rounding and data processing.

S4.1. Anhydrous Blend

The radionuclide (Rn) content in blends was calculated from the expression:

$$A_{Rn(blend)} = P_{OPC} \cdot A_{Rn(OPC)} + P_{FA} \cdot A_{Rn(FA)} \quad (1)$$

where:

$A_{Rn(blend)}$ = radionuclide activity concentration (in Bq·kg^{-1}) in the blend

$A_{Rn(OPC)}$ = radionuclide activity concentration (in Bq·kg^{-1}) in the OPC

$A_{Rn(FA)}$ = radionuclide activity concentration (in Bq·kg^{-1}) in the FA

P_{OPC} = percentage of OPC in the blend (OPC + FA)

P_{FA} = percentage of FA in the blend (OPC + FA)

for instance, for ^{40}K in a 95 % OPC / 5 % FA blend:

$$A_{40_K(blend)} = P_{OPC} \cdot A_{40_K(OPC)} + P_{FA} \cdot A_{40_K(FA)} \quad (2)$$

In the example: $A_{40_K(blend)} = 0.95 \cdot 233 + 0.05 \cdot 292 = 236 Bq \cdot kg^{-1}$ (3)

Uncertainty: $u(A_{Rn(blend)}) = \sqrt{P_{OPC}^2 \cdot u^2(A_{Rn(OPC)}) + P_{FA}^2 \cdot u^2(A_{Rn(FA)})}$ (4)

where:

$u(A_{Rn(blend)}) =$ activity concentration uncertainty for the radionuclide in the blend

$u(A_{Rn(OPC)}) =$ activity concentration uncertainty for the radionuclide in OPC

$u(A_{Rn(FA)}) =$ activity concentration uncertainty for the radionuclide in FA

for instance, for ^{40}K in a 95 % OPC / 5 % FA blend:

$$u(A_{40_K(blend)}) = \sqrt{P_{OPC}^2 \cdot u^2(A_{40_K(OPC)}) + P_{FA}^2 \cdot u^2(A_{40_K(FA)})} \quad (5)$$

In the example:

$$u(A_{40_K(blend)}) = \sqrt{0.95^2 \cdot 15^2 + 0.05^2 \cdot 18^2} = 14.3 Bq \cdot kg^{-1} \quad (6)$$

S4.2. Fresh Paste Prepared with Liquid/Solid (l/s) Ratio Used in the Paper

Theoretical calculation for OPC + fly ash hydrated paste, assuming all water to be taken up in the blend.

$$A_{Rn(blend\_hydrated)} = (P_{OPC} \cdot A_{Rn(OPC)} + P_{FA} \cdot A_{Rn(FA)}) \cdot P_{(blend\_hydrated)} \quad (7)$$

where:

$A_{Rn(fresh\ blend)}$= radionuclide (Rn) activity concentration in the fresh blended paste

$P_{Rn(fresh\ blend)}$= percentage of radionuclide in fresh blended paste

$$P_{Rn(blend\_hydrated)} = \left(\frac{1}{1 + l/s}\right) \quad (8)$$

In the example:
l/s ratio = 0.32

$$P_{(5\%FA+95\%OPC)\_hydrated)} = \left(\frac{1}{1+l/s}\right) = \left(\frac{1}{1+0.32}\right) = 0.757 \quad (9)$$

where l/s = liquid solid ratio used to prepare the cement (OPC) or blended (OPC + FA) paste

$$A_{40_K(blend\_hydrated)} = (P_{OPC} \cdot A_{40_K(OPC)} + P_{FA} \cdot A_{40_K(FA)}) \cdot P_{40_K(5\%FA+95\%OPC)\_hydrated)} \quad (10)$$

In the example:

$$A_{40_K(blend\_hydrated)} = (0.95 \ \cdot 233 + 0.05 \cdot 292) \cdot 0.757 = 178.8 Bq \cdot kg^{-1} \quad (11)$$

Uncertainty:
Assuming l/s uncertainty to be 0 or negligible compared to activity concentration uncertainty:
$u(A_{Rn(fresh\ blend)})$ = uncertainty of radionuclide (Rn) activity concentration after hydration

$$u(A_{Rn(blend\_hydrated)}) = \sqrt{P^2_{Rn(blend\_hydrated)} \ \left(P^2_{OPC} \cdot u^2(A_{Rn(OPC)}) + P^2_{FA} \cdot u^2(A_{Rn(FA)})\right)} \quad (12)$$

$$u(A_{Rn(blend\_hydrated)}) = \sqrt{P^2_{Rn(blend\_hydrated)} \ \ u^2(A_{Rn(blend)})} \quad (13)$$

In the example:

$$u(A_{40_K(blend\_hydrated)}) = \sqrt{P^2_{40_K(blend\_hydrated)} \ \ u^2(A_{40_K(blend)})} = P_{40_K(blend\_hydrated)} \ \ u(A_{40_K(blend)}) \quad (14)$$

$$u(A_{40_K(blend\_hydrated)}) = 0.757 \cdot 14.3 = 10.8 Bq \cdot kg^{-1} \quad (15)$$

S4.3. Fresh and Dry (Unbound Water Loss) Blended Paste

Assuming unbound water loss in the fresh paste to be equal to the maximum experimental value.
During drying, the 95 % OPC + 5%FA paste prepared at an l/s ratio = 0.32 would lose 6.25 % of water/mass, calculated as:

$$A_{Rn(blend\_hydrated)water\_loss} = \left(\frac{A_{Rn(blend\_hydrated)}}{(1-P_{waterloss})}\right) \quad (16)$$

where:

$A_{Rn(blend\_hydrated)water\_loss}$= activity concentration of the radionuclides Rn after a maximum dryness of the pastes.
In this example 6.25 % of the 'water' was assumed to be unbound and liable to post-paste hydration loss:

$$A_{40_K(blend\_hydrated)water\_loss} = \left(\frac{A_{40_K(blend\_hydrated)}}{(1 - P_{waterloss})}\right) \quad (17)$$

In the example:

$$A_{40_K(blend\_hydrated)water\_loss} = \left(\frac{178.8}{(1 - 0.0625)}\right) = 190.7 Bq \cdot kg^{-1} \quad (18)$$

Uncertainty
$\left(A_{Rn(fresh\ blend)water_{loss}}\right)$ = radionuclide (Rn) activity concentration uncertainty for specimens dried to a constant weight

$$u(A_{Rn(blend\_hydrated)water\_loss}) = \left(\frac{1}{(1 - P_{waterloss})}\right) \cdot u(A_{Rn(blend\_hydrated)}) \quad (19)$$

In the example:

$$u(A_{40_K(blend\_hydrated)water\_loss}) = \left(\frac{1}{(1 - P_{waterloss})}\right) \cdot u(A_{40_K(blend\_hydrated)}) \quad (20)$$

Therefore:

$$u\left(A_{40_K(fresh\ blend)water_{loss}}\right) = \left(\frac{1}{(1 - 0.0625)}\right) \cdot 10.8 = 11.5\ \ Bq \cdot kg^{-1} \quad (21)$$

ACI uncertainty is described in (1).

$$ACI = \frac{A_{40_K}}{3000} + \frac{A_{226_{Ra}}}{300} + \frac{A_{212_{Pb}}}{200} \quad (22)$$

In the example:

$$ACI = \frac{236}{3000} + \frac{26}{300} + \frac{23.4}{200} = 0.282 \quad (23)$$

Assuming activity concentration uncertainty only to the exclusion of model uncertainty for 3000, 300 and 200

$$u(ACI) = \sqrt{\left(\frac{1}{3000}\right)^2 u^2(A_{40_K}) + \left(\frac{1}{300}\right)^2 u^2(A_{226_{Ra}}) + \left(\frac{1}{200}\right)^2 u^2(A_{212_{Pb}})} \quad (24)$$

$u(ACI) =$ ACI uncertainty

In the example:

$$u(ACI) = \sqrt{\left(\frac{1}{3000}\right)^2 15^2 + \left(\frac{1}{300}\right)^2 2.5^2 + \left(\frac{1}{200}\right)^2 2.4^2} = 0.015 \tag{25}$$

These calculations were performed for all paste types and radionuclide activity concentrations using VBA (Excel) software.

S4.4. Uncertainties for Experimental Values

The results observed in Figure 4 were found using the means of replicated measurements or samples.

The weighted mean activity concentration for two experimental values factoring in their respective uncertainties is defined as:

$$\overline{A}_{Rn} = \frac{\sum_{i=1}^{N} A_{Rn(i)} \cdot \sum_{i=1}^{N} u(A_{Rn(i)})}{\sum_{i=1}^{N} u(A_{Rn(i)})} \tag{26}$$

where:

$\overline{A}_{Rn} =$ mean radionuclide (Rn) activity concentration

$A_{Rn(i)}$= i-th sample or measurement activity concentration

$u(A_{Rn(i)})$= uncertainty of i-th radionuclide activity concentration

The uncertainty of the weighted average was found further to the Bambynek criterion [39], i.e., as the higher of the external ($u(\overline{A})_{ext}$) or internal ($u(\overline{A})_{int}$) uncertainty, both calculated for a coverage factor k = 1.

$$u(\overline{X})_{ext} = \sqrt{\frac{\sum_{i=1}^{N} u(A_{Rn(i)})_i \cdot \left(A_{Rn(i)} - \overline{A}_{Rn}\right)^2}{\frac{N}{N-1} \cdot \sum_{i=1}^{N} u(A_{Rn(i)})_i}} \tag{27}$$

materials

1
